# Photonic Dirac cavities with spatially varying mass term

**DOI:** 10.1126/sciadv.abq4243

**Published:** 2023-03-22

**Authors:** Kai Chen, Filipp Komissarenko, Daria Smirnova, Anton Vakulenko, Svetlana Kiriushechkina, Irina Volkovskaya, Sriram Guddala, Vinod Menon, Andrea Alù, Alexander B. Khanikaev

**Affiliations:** ^1^ Electrical Engineering and Physics, The City College of New York (USA), New York, NY 10031, USA.; ^2^ Department of Physics, City College of New York, New York, NY 10031, USA.; ^3^ Physics Program, Graduate Center of the City University of New York, New York, NY 10016, USA.; ^4^ Research School of Physics, Australian National University, Canberra ACT 2601, Australia.

## Abstract

In recent years, photonics has proven itself as an excellent platform for emulation of relativistic phenomena. Here, we show an example of relativistic-like trapping in photonic system that realizes Dirac-like dispersion with spatially inhomogeneous mass term. The modes trapped by such cavities, their energy levels, and corresponding orbitals are then characterized through optical imaging in real and momentum space. The fabricated cavities host a hierarchy of photonic modes with distinct radiation profiles directly analogous to various atomic orbitals endowed with unique characteristics, such as pseudo-particle-hall symmetry and spin degeneracy, and they carry topological charge which gives rise to radiative profiles with angular momentum. We demonstrate that these modes can be directionally excited by pseudo-spin–polarized boundary states. In addition to the fundamental interest in the structure of these pseudo-relativistic orbitals, the proposed system offers a route for designing new types of nanophotonic devices, spin-full resonators and topological light sources compatible with integrated photonics platforms.

## Teaser

Silicon photonic nanostructures with massive Dirac dispersion offer an opportunity for emulating relativistic trapping of light.

## INTRODUCTION

Photonics has established itself as a powerful experimental platform to study complex physical systems and mimic unattainable physical settings over a table-top platform (*1*, *2*) (*3*–*18*). Examples of physical systems that were successfully emulated in photonics include those described by the Dirac equation, spanning from two-dimensional anomalous quantum-Hall, quantum spin-Hall, and valley-Hall phases to three-dimensional topological crystals and even synthetic and higher dimensions beyond the limitations of physical space (*19*–*28*). Moreover, photonics not only enables experimenting within these highly complex physical systems but can also enrich the emulated physics by introducing active control (*29*, *30*), non-Hermiticity, and nonlinear effects (*31*–*36*) that can be used to mimic multiparticle interactions. Light-matter interactions can also endow the problem with multiphysics features, such as the formation of hybrid states of optical and solid-state excitations in polaritonic Dirac systems (*37*–*43*).

In this work, we explore an innovative application of Dirac-analog photonic systems to emulate relativistic-like trapping. Besides the fundamental aspects, the proposed Dirac trapping potentials represent spin-degenerate artificial optical resonators and therefore are compatible with spin-full topological photonic structures, such as spin-Hall systems in two dimensions.

## RESULTS

### Theoretical model of the Dirac cavity

The Dirac equation describes charged spin-1/2 relativistic particles, such as electrons and positrons, interacting with an electromagnetic field. This interaction can be written in the form of coupled equations for the positive and negative energy spinors (particles and antiparticles)

$$\left([\begin{array}{cc} mc^{2}-E+e\phi & c\text{σ}\times (p-eA)\\ -c\text{σ}\times (p-eA) & mc^{2}+E-e\phi \end{array}\right]\left(\begin{array}{c} \psi_{+}\\ \psi_{-} \end{array}\right)=0$$ (1)

where *m* is the particle mass, ℰ and **p** are the particle energy and momentum, (ϕ/*c*, − **A**) is Minkowski four-vector potential, and ψ_±_ represents particle (antiparticle) 2-spinor. We adopt a dimensionless notation with speed *c* = 1 and elementary charge *e* = 1. Equation 1 supports an energy spectrum in the form $E=\pm \sqrt{m^{2}+{|p-A|}^{2}}+e\phi$.

One can see from Eq. 1 that no scalar electric Coulomb potential ϕ can trap a massless relativistic particle. For massive particles, trapping with Coulomb potentials is also limited as is can trap only either electrons (particles) or positrons (holes) but not both. Last, the potential ϕ unevenly affects the spectrum of electrons and positrons, thus breaking particle-hole symmetry. To circumvent these limitations, here, we explore a different confinement mechanism via spatially varying mass term. The introduction of a mass term gaps the Dirac spectrum and thus allows confined solutions whenever there is a region of lower mass *m_a_* surrounded by a region of larger mass *m_b_* (background mass), i.e., *m_a_* < *m_b_*. While the background region has solutions with a continuous spectrum, the variation of the mass term produces an effective trapping mechanism, which gives rise to discrete localized states.

**Fig. 1.**
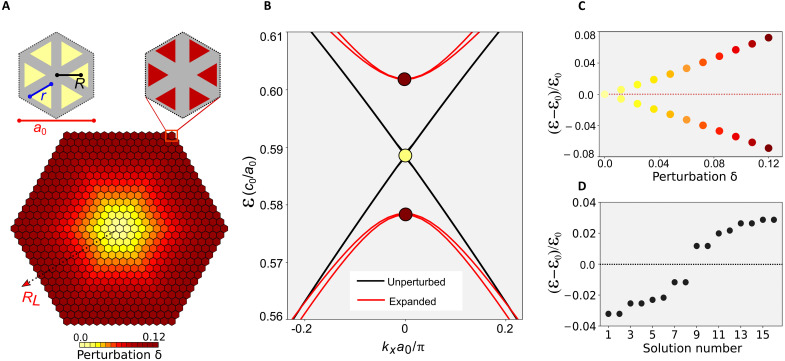
Spin-degenerate trapping with spatially variable mass term as a Dirac cavity. (**A**) Sketch of the photonic Dirac cavity and its unit cells, with parameters $R=\frac{a_{0}}{3}\;(1+\delta )$ and $r=\frac{1.19a_{0}}{3}\;(1-3.4\delta^{2})$, where *a*_0_ is the lattice constant. The Dirac cavity is created in a finite-thickness nanostructured silicon metasurface. (**B**) Simulated photonic band structure of uniform metasurfaces: gapless (black lines, δ = 0) and gapped (red curves, δ = 0.05). (**C**) Degenerate eigenfrequencies [denoted by dark red points in (B)] at Γ point as a function of the expansion strength δ, in units of $\varepsilon_{0}=\frac{c_{0}}{a_{0}}$, which is the eigenfrequency of the Dirac point at *k* = 0 [denoted by the yellow point in (B)]. (**D**) Typical spectrum of bound states for the Dirac cavity calculated with the COMSOL simulation.

As a platform for experimental realization, we use spin-degenerate photonic crystals with Dirac dispersion (*44*) and introduce a position-dependent mass term via lattice symmetry reduction. The proposed system consists of hexamers of triangle-shaped holes arranged into a triangular lattice (Fig. 1A). Spatially varying geometric parameters of the lattice emulates the variation of the mass term and ensures that the effective particle-hole symmetry is retained across the structure. The effective optical response of spin-degenerate photonic crystals based on a triangular lattice of hexamers is captured by the two-dimensional Dirac Hamiltonian (*18*, *45*, *46*).

$${\hat{H}}_{↑↓}(k)=\mu (k){\hat{\sigma }}_{z}\mp k_{x}{\hat{\sigma }}_{x}-k_{y}{\hat{\sigma }}_{y}$$ (2)

where ↑ (↓) denotes the pseudo–spin-up (pseudo–spin-down) state, μ(**k**) = *m* + *Bk*^2^ is a dispersive mass, **k** = {*k_x_*, *k_y_*} is the wave vector, and ${\hat{\sigma }}_{i}$ are Pauli matrices. At the Γ point of the Brillouin zone (**k** = 0), the eigenfrequencies of doubly degenerate dipolar and quadrupolar states are ℰ = ± *m*, where the mass term *m* depends on the strength of the expansion introduced in the unit-cell hexamers and quantified by δ (Fig. 1A). Without expansion, i.e., for δ = 0 (and *R* = *a*_0_/3), where δ is the distortion parameter, the doubly degenerate Dirac cones appear at the *K* and *K*′ points in the Brillouin zone of the honeycomb lattice. If the distortion is introduced, i.e., for δ ≠ 0, then the Dirac bands are folded to form a doubly degenerate spectrum at the Γ point, as shown in Fig. 1B, and the low-energy effective theory (Eq. 2) corresponds to a massive Dirac particle with pseudo–spin-up and pseudo–spin-down components.

By adjusting our photonic crystal through the degree of expansion δ (see Fig. 1C), we can design a system with the mass term *m*(*x*, *y*) that gradually varies in space. To implement rotationally symmetric trapping cavities, the mass term profiles satisfy $m(x,y)=m({\hat{R}}_{z}x,{\hat{R}}_{z}y)$, where ${\hat{R}}_{z}$ is a continuous or discrete rotational operation around the *z* axis. We chose the position-dependent mass term *m*(*x*, *y*) = α [1 − *e*^−β(*x*^2^+*y*^2^)^], where α and β determine the depth and slope of the effective mass term. Because our photonic crystal preserves *C*_6_ rotational symmetry, the mass term is a function of layers, i.e., $m(R_{L})\equiv \alpha \;(1-e^{-\beta R_{L}^{2}})$, with *R_L_* corresponding to the radius of the *L*th layer around a given center of the cavity). In our photonic realization, $R_{L}=\frac{a_{0}}{3}(1+\delta )$, where *a*_0_ is the lattice constant and δ varies from 0 to 0.12 (see Fig. 1A). We note that the choice of this “smooth” Gaussian-like mass term profile is justified by the additional opportunities to control spectrum, trapping, spin degeneracy, and even radiative properties of the cavity. As shown in section S5, smoother mass-term profile allows reducing the effect of spin mixing and leads to higher quality factor (longer lifetime) orbitals of the system. Additional theoretical analysis based on the tight binding model can also be found in section S3.

One important property of the Dirac equation, stemming from particle-hole symmetry, is the symmetry of the energy spectrum with respect to zero energy level, and in our structure, we aim to preserve this symmetry. However, for a photonic crystal with a fixed size of holes, this symmetry in spectrum with respect to “zero energy” (the Dirac point frequency ℰ_0_) is broken, i.e., ℰ_+_(δ) + ℰ_−_(δ) ≠ ℰ_0_. To address this issue, we also adjust the size of holes for each cavity layer to ensure that the condition ℰ_+_(δ) + ℰ_−_(δ) = ℰ_0_ is satisfied (Fig. 1C). Figure 1D shows the spectrum of the resultant cavity with clear presence of discrete states with energies symmetric with respect to the zero energy level.

While the effective mass term of our photonic crystal satisfies *C*_6_ rotational symmetry, to gain some insight in this system, we consider a continuous limit, in which case the solutions of the Dirac Eq. 2 can be approximated by the solutions with mass term that satisfies continuous rotational symmetry (see sections S1 and S2 for details). We adopt polar coordinates and, assuming adiabatic variation of the mass term, neglect all second-order space derivatives in μ(**k**) for simplicity. Since the Hamiltonian (Eq. 2) commutes with the *z* component of the total angular momentum ${\hat{J}}_{z}=-i\partial_{\varphi }\pm \frac{1}{2}{\hat{\sigma }}_{z}$, the two-component eigenvectors for spin-up and spin-down states can be labeled by the quantum numbers and have the form

$$|\psi_{↑/↓}(\rho ,\varphi )>=\frac{1}{\sqrt{2\pi \rho }}\left[\begin{array}{c} f_{L_{↑/↓}}(\rho )e^{\pm i\varphi }\\ \mp {ig}_{L_{↑/↓}}(\rho ) \end{array}\right]e^{iL_{↑/↓}\varphi }$$ (3)

where *L*_↑/↓_ = 0, ± 1, ± 2… is the quantum number of the orbital angular momentum ${\hat{L}}_{z}=-i\partial_{\varphi }$. The Hamiltonian (Eq. 2) acting on the column-vector (*f*_*L*_↑/↓__, *g*_*L*_↑/↓__)*^T^* in (Eq. 3) can be expressed as

$${\hat{H}}_{↑/↓}=m(\rho ){\hat{\sigma }}_{z}+i\partial_{\rho }{\hat{\sigma }}_{y}\mp \frac{J_{↑/↓}}{\rho }{\hat{\sigma }}_{x}$$ (4)

where *J*_↑/↓_ = *L*_↑/↓_ ± 1/2. The first term in Eq. 4 denotes the position-dependent mass term: The mass is positive for a particle, while the mass of a hole (antiparticle) takes a negative value. The other two terms depend on the quantum number *J*_↑/↓_ and the pseudo-spin. The eigenvalue problem for each spin obeys particle-hole symmetry ℰ(1 − *L*_↑/↓_) = −ℰ(*L*_↑/↓_) with interchange *f*(1 − *L*_↑/↓_) ↔ *g*(*L*_↑/↓_), *g*(1 − *L*_↑/↓_) ↔ *f*(*L*_↑/↓_). This means that for each mode with “positive energy” eigenvalue ℰ, there exists a “negative energy” partner of opposite frequency ( –ℰ) with same intensity distribution. Depending on the trapping cavity size, there may be several modes with the same *L*_↑/↓_ but different radial distribution. Similar to a hydrogen atom problem, these distinct radial solutions can be labeled by an additional quantum number *n*, which is the number of nodes in the orbital in the radial direction.

### Experimental realization

**Fig. 2.**
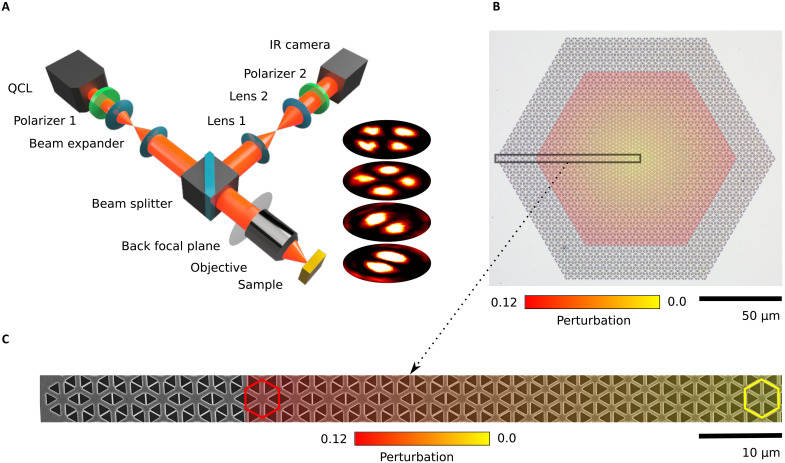
Experimental setup, optical, and scanning electron microscopy image of the photonic Dirac cavity. (**A**) Scheme of experimental setup used to perform imaging of the modes of Dirac cavity in Fourier and real-space domains. (**B**) Optical image of fabricated Dirac cavity. (**C**) Scanning electron microscopy image of highlighted area in Fig. 3B, which reveals varying geometry of unit cells with *a*_0_ = 4.08 μm. Unperturbed unit cell is depicted by yellow hexagon, and the most expanded unit cell is depicted by red hexagon.

To demonstrate our photonic Dirac cavity experimentally, we fabricated the silicon photonic-crystal metasurface with varying mass term as shown in Fig. 2. A silicon-on-sapphire (SOS) substrate with 1-μm-thick silicon layer was patterned using electron beam lithography, followed by anisotropic reactive ion plasma etching process (see Materials and Methods for details). While lithography gives rise to slight rounding of corners of the triangular holes, this, however, did not lead to any substantial modification of the photonic properties (section S6). The design of the structure was as in Fig. 1A, and six additional layers of the most expanded unit cells surrounding the cavity were added as a uniform background medium. The addition of background layers has no direct effect on the cavity modes but ensures that the signal collected with the optical objective from the structure is dominated by scattering from the modes themselves and is not overshadowed by scattering between patterned and unpatterned silicon.

**Fig. 3.**
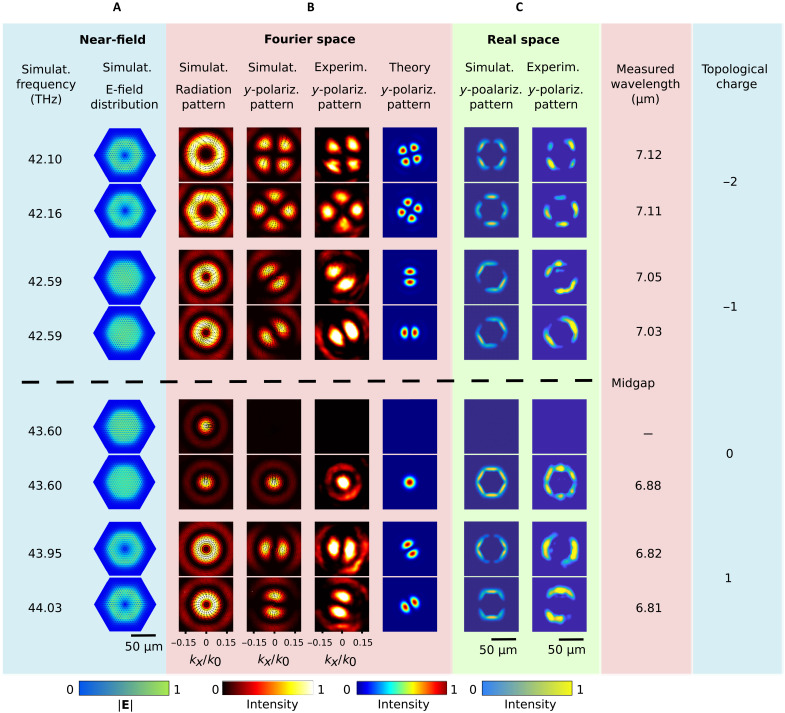
Modal profiles of the photonic Dirac cavity. (**A**) Electric field ∣**E**∣ distribution of the low-order eigenmodes attained in the first-principles simulations. The dashed line shows a midgap frequency level. (**B** and **C**) Profiles of the modes in Fourier space (B) and real space (C). Resonant frequencies of the modes and their topological charge are given in the two rightmost columns.

A custom-built mid–infrared (IR) microscope (*39*), equipped with thermal imaging camera and quantum cascade laser (QCL) (Fig. 2A), was used to image modes of the Dirac cavity in both real- and Fourier-space domains (see Materials and Methods for details) in the spectral range from ~6.5 to ~7.6 μm with a spectral resolution of 5 nm. The orbitals of the Dirac cavity were characterized in cross-polarization for linearly polarized excitation, while the spot size of a laser beam was comparable with the full structure size. Figure 3 shows experimental results for a few lowest-order modes of the structure alongside with the results of full-wave numerical simulations. The extended table of the modes is presented in S4 fig. S5. The first column in Fig. 3A shows the simulated near-field distributions of the nearly degenerate mode pairs around the midgap. We classify the modes according to the radiation pattern and polarization texture, shown in the second column.

The far-field mode profiles were found to exhibit almost axially symmetric intensity distributions, reminiscent of the cylindrical vector beams. The observed far-field polarization can be reconstructed from the right and left circular contributions corresponding to the dipolar component in the Fourier-transformed wave functions of the opposite spins at degenerate frequency levels ℰ_↓_(*L*_↓_) = ℰ_↑_(*L*_↑_) with *L*_↓_ = −*L*_↑_. Its angular dependence can be inferred from Eq. 3

$$E_{\mathrm{FF}}\propto e^{il\varphi }(x_{0}-iy_{0})\pm e^{-il\varphi }(x_{0}+iy_{0})$$ (5)

where *l* = *L*_↓_ − 1 = −*L*_↑_ − 1 is the topological charge and **E** is the electric field of the mode. In Fig. 3, we recognize polarization distributions of linearly polarized (*l* = 0), radially and azimuthally polarized (*l* = 1), and hybridly polarized (*l* = −1) beams. At *l* ≠ 0, the beam hosts a phase singularity and has a doughnut-like shape. Through the polarizer, the doughnut is transformed into lobes. The theoretical modes derived from the continuum Dirac model correspond to *L*_↓_ = 2,1,0, and −1, as the wavelength increases from the bottom of Fig. 3. The real-space images in Fig. 3C, obtained in the far field, correspond to the spatial distribution of the mode intensity near the surface of the structure, keeping in mind that the objective only collects radiating waves.

**Fig. 4.**
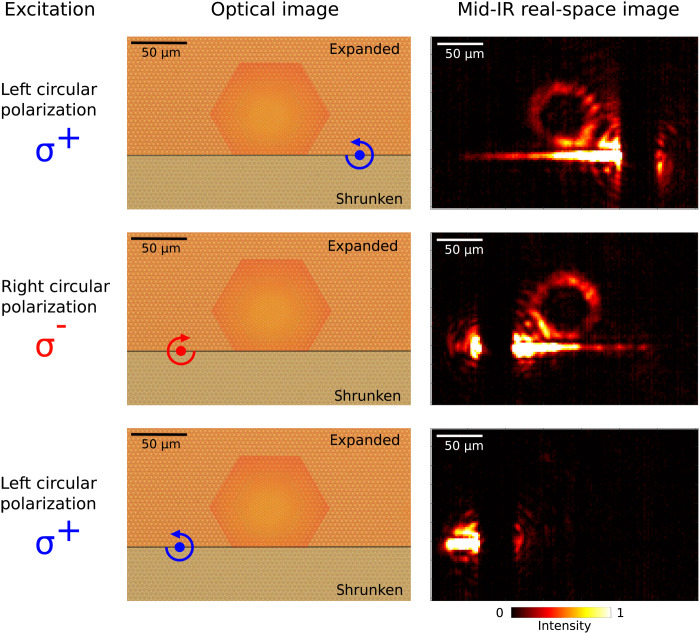
Spin-polarized edge state driven excitation of the Dirac cavity. Excitation of the photonic Dirac cavity by side-coupling to the edge state guided by domain wall between topological (expanded) and trivial (shrunken) domains. The left column shows optical images of the structure with the Dirac cavity, domain wall, and excitation points depicted in color. The right column shows corresponding real-space mid-IR images, all obtained at the same frequency matching the first cavity mode below the midgap frequency.

Our experimental results demonstrate the narrow-band character of bound modes and their spatial confinement, as well as the predicted symmetry of both spatial and angular patterns of Dirac cavity orbitals. Obtained by direct and back focal plane imaging, the intensity profiles confirm the existence of a hierarchy of spatially confined modes of the massive Dirac Hamiltonian with a varying mass term and show excellent agreement with both first-principles simulations and our analytical model. We would also like to note that our approach to trapping relies purely on the variation of the mass term, and other interesting mechanisms of confinement in Dirac systems, due to the modal mismatch of bulk states between topological and trivial domains (*47*) and the creation of two-dimensional winding with two mass terms (*26*, *48*), have been proposed recently.

### Spin-selective excitation via evanescent coupling to helical edge states

The proposed Dirac cavities have an interesting property of spin degeneracy, which ensures that the trapped states appear to be doubly degenerate (up to a small perturbation caused by the spin mixing; see section S1). Moreover, the topological charge, defining the chirality of structured light described by Eq. 5 and emitted by the cavity, is directly linked to the value of the pseudo-spin. Thus, the spin-polarized excitation of the cavity modes opens an opportunity to excite them selectively and thus control the field radiated by the cavity. To achieve this spin-selective excitation, one can simply excite the modes by the circularly polarized incident light and rely on the conservation of the angular momentum, as it was done in the experiments above. Another more interesting way for spin-selective coupling is to exploit the underlying topological nature of the system and couple the Dirac cavity modes to the spin-polarized boundary modes, which can be produced in our structure by adding a domain wall into the structure. The domain wall created by the abrupt reversal of the perturbation of the structure from shrunken to expanded gives rise to the reversal of the mass term and emergence of the spin-polarized edge states. Providing that the domain wall is created in close proximity to the Dirac cavity, the edge modes will directly couple to the cavity modes of the same spin. Moreover, the one-way character of the edge states offers opportunity for the directional excitation of the cavity modes.

To experimentally demonstrate the possibility of this directional spin-polarized excitation, we have optimized the distance between the Dirac cavity and the topological domain wall to attain desirable coupling of the boundary mode to the first spin-degenerate mode below the midgap frequency. The optimized structure was then fabricated in the SOS substrate (Fig. 4) and characterized in the far field by direct excitation of the edge mode by circularly polarized light focused on the domain wall. As can be seen from Fig. 4, this circularly polarized excitation gives rise to the directional excitation of the oppositely propagating forward spin-up and backward spin-down edge modes. We observed that the edge modes side-couple to the cavity mode leading to the spin-selective directional excitation (Fig. 4, top and middle) for left and right circularly polarized excitation carried out from the left and right sides of the cavity. As expected, the excitation with the “wrong” pseudo-spin carried the mode away from the cavity without excitation of the mode (Fig. 4, bottom).

## DISCUSSION

In this work, we realized a photonic crystal with a spatially variable mass term, demonstrating a new type of photonic cavities emulating relativistic-like, spin-degenerate, and particle-hole symmetric trapping. We fabricated and performed optical characterization of a prototype device operating in the mid-IR region. Direct imaging of the structure in both real and Fourier spaces revealed a hierarchy of photonic modes of different orbitals were directly linked to the physics captured by our effective Dirac model. The modes of the cavities were found to have a rich structure with their vorticity related to the topological charge of the modes. We also investigated coupling of the cavity to spin-polarized edge states and demonstrated spin-selective directional excitation of the cavity modes. In future research our Dirac cavities, emulating relativistic trapping, effective “Dirac atoms,” can also be spatially arranged to form spin-degenerate and particle-hole symmetric arrays of “molecules” or even crystals that may exhibit even more exciting physics properties stemming from the collective response. Complementing to other ways of trapping in Dirac systems (*49*, *50*), our work suggests that optical spin-full metasurfaces with Dirac dispersion can serve as an efficient toolkit to probe relativistic wave physics on a silicon chip and also envisions applications, such as source of arbitrary vector beams for on-chip generation of structured light.

## MATERIALS AND METHODS

### Sample fabrication

The Dirac cavity structure was fabricated by imprinting an array of triangular-shaped holes with varying expansion ratio and size into SOS substrates (1 μm of Si and 500 μm of sapphire) by means of electron beam lithography (Elionix ELS-G100). First, 300-nm-thick layer of e-beam resist ZEP520A was spin-coated on substrate with 4-min baking at 180°C. After that, to prevent charging during electron-beam exposure, a 15-nm-thick gold film was sputtered on top of the resist. Electron-beam lithography exposure was followed by gold etch. Development of the resist was carried out in *n*-amyl acetate at 0°C for 1 min. Next, the Oxford PlasmaPro System Inductively Coupled Plasma was used for the anisotropic plasma etching of silicon for the full 1-μm depth by a recipe based on C_4_F_8_/SF_6_ gasses with etching rate of about 2.5 nm/s. During the last stage, the unexposed resist was removed by N-methyl-2-pyrrolidone (NMP) solution heated to 60°С.

### Experimental setup

For collecting modes profiles of the fabricated nanophotonic Dirac atom, we have built a mid-IR–range operating microscope based on a Daylight MIRcat-QT QCL and INO MicroXcam-384-i camera. The setup was designed for imaging a sample by a refractive objective in both real space and Fourier space at fixed frequencies (the so-called isofrequency contours) in the reflection geometry with normal incidence of a laser beam. By tuning the wavelength of QCL, we acquired the isofrequency contours within the spectral range of interest with a fine step (5 nm). The laser beam of the QCL laser was narrowed down with a 20-cm CaF_2_ lens and directed through ZnSe 50/50 beam splitter. We used a Thorlabs Black Diamond molded aspheric lens with 0.56 numerical aperture as an objective. Excitation area on the sample was comparable with the sample size (150 μm). Radiation patterns of the observed modes were collected using the back focal plane imaging technique. While the back focal plane of the objective contains angular distribution of radiation collected by that objective, the far-field directionality diagram of the structure’s emission can be recovered from it. This technique was implemented using a set of two 20-cm focal length CaF_2_ lenses, which images a back focal plane of the operated objective on the IR camera’s plane in 4*f* configuration. For direct real-space imaging at the same setup, the second lens was replaced by the other one with *f* = 10 cm to bring an image of the sample onto the camera’s plane. A wire grid polarizer was used for implementing crossed polarization configuration, which allowed us to suppress a background reflection from the sample. Sequential tuning of the QCL wavelength along with recording real- and Fourier-space images allowed us to collect angular and spatial profiles of the photonic modes residing within the spectral range from 6.5 to 7.6 μm.

## Supplementary Materials

### This PDF file includes:

Sections S1 to S6 Figs. S1 to S9
