# Supplementary material for: Photonic Dirac cavities with spatially varying mass term
Source: Sci Adv. 2023 Mar 22;9(12):eabq4243. doi: 10.1126/sciadv.abq4243 (PMC10032596; doi:10.1126/sciadv.abq4243)
Supplement: Supplementary file 1 — Sections S1 to S6 Figs. S1 to S9 [file sciadv.abq4243_sm.pdf]

Science Advances
AAAS

Supplementary Materials for

Photonic Dirac cavities with spatially varying mass term

Kai Chen *et al.*

Corresponding author: Alexander B. Khanikaev, akhanikaev@ccny.cuny.edu

Sci. Adv. **9**, eabq4243 (2023)
DOI: 10.1126/sciadv.abq4243

This PDF file includes:

Sections SA to SF
Figs. S1 to S9

Supplementary Materials

Section 1: Solutions of the continuum model

In this section, we analyze solutions of the continuum Dirac model with the position dependent mass term satisfying the rotational symmetry. For the two-dimensional system, the effective continuum Hamiltonian takes the form

$$\hat{H}_{\text{eff}}(\boldsymbol{k}) = \begin{pmatrix} \hat{H}_{\uparrow}(\boldsymbol{k}) & 0 \\ 0 & \hat{H}_{\downarrow}(\boldsymbol{k}) \end{pmatrix}, \tag{S1}$$

where $\hat{H}_{\uparrow}(\boldsymbol{k}) = m(\boldsymbol{r})\hat{\sigma}_z + k_x\hat{\sigma}_x + k_y\hat{\sigma}_y$ and $\hat{H}_{\downarrow}(\boldsymbol{k}) = m(\boldsymbol{r})\hat{\sigma}_z - k_x\hat{\sigma}_x + k_y\hat{\sigma}_y$. For a circularly symmetric problem, the mass profile $m(\mathbf{r}) = m(\rho)$ is a radial function in the polar coordinate system (ρ, φ). We consider a stepwise case in the geometry, where a circular domain $\rho < R$ with zero mass $m = 0$ is surrounded by the insulator with the positive mass $m = M > 0$.

The time evolution equation with the Hamiltonian Eq. (S1) can be formulated for two spin blocks

$$\begin{aligned} i\frac{\partial \Psi_1}{\partial t} &= \pm e^{\pm i\varphi}\left(i\frac{\partial}{\partial \rho} \mp \frac{1}{\rho}\frac{\partial}{\partial \varphi}\right)\Psi_2 + M\Psi_1 , \\ i\frac{\partial \Psi_2}{\partial t} &= \pm e^{\mp i\varphi}\left(i\frac{\partial}{\partial \rho} \pm \frac{1}{\rho}\frac{\partial}{\partial \varphi}\right)\Psi_1 - M\Psi_2 , \end{aligned} \tag{S2}$$

where the upper and lower signs correspond to the spin-up and spin-down states, respectively.

System Eq. (S1) possesses solutions with harmonic time dependence and radial symmetry

$$\begin{pmatrix} \Psi_1 \\ \Psi_2 \end{pmatrix}(\rho, \varphi, t; L) = \begin{pmatrix} \psi_1 \\ \psi_2 \end{pmatrix} e^{-i\omega_n t} = e^{-i\omega_n t}\begin{pmatrix} \bar{f}e^{\pm i\varphi} \\ \mp i\bar{g} \end{pmatrix} e^{iL\varphi} , \tag{S3}$$

where $L = 0, \pm 1, \pm 2 \ldots$ is the azimuthal number. The eigenvalue problem $\hat{H}_{\uparrow\downarrow}\boldsymbol{\psi} = \omega_n\boldsymbol{\psi}$ in the polar coordinate system reads

$$\omega_n \begin{pmatrix} \psi_1 \\ \psi_2 \end{pmatrix} = \begin{pmatrix} M & \pm i e^{\pm i\varphi}\left(\frac{\partial}{\partial \rho} \pm \frac{i}{\rho}\frac{\partial}{\partial \varphi}\right) \\ \pm i e^{\mp i\varphi}\left(\frac{\partial}{\partial \rho} \mp \frac{i}{\rho}\frac{\partial}{\partial \varphi}\right) & -M \end{pmatrix}\begin{pmatrix} \psi_1 \\ \psi_2 \end{pmatrix} . \tag{S4}$$

The spectrum yielded by Eq. (S4) is discrete. The radial functions $\bar{f}(\rho)$ and $\bar{g}(\rho)$ for the spin down obey the following coupled ordinary differential equations:

$$\begin{aligned} \left(\frac{d}{d\rho} + \frac{L}{\rho}\right)\bar{g} + (M - \omega_n)\bar{f} &= 0 , \\ \left(\frac{d}{d\rho} - \frac{L-1}{\rho}\right)\bar{f} + (M + \omega_n)\bar{g} &= 0 . \end{aligned} \tag{S5}$$

These equations have a symmetry with respect to the following substitution $\bar{f}_L \leftrightarrow \bar{g}_L$, $\omega_n \leftrightarrow -\omega_n$, $L \leftrightarrow (1 - L)$. At the same frequency but for the opposite spin (spin up) the angular momentum is opposite, $L_\uparrow = -L_\downarrow$.

From Eqs. (S5), we obtain a second-order differential equation for $\bar{g}$:

$$\frac{d^2\bar{g}}{d\rho^2} + \frac{1}{\rho}\frac{d\bar{g}}{d\rho} + \left({\omega_n}^2 - M^2 - \frac{L^2}{\rho^2}\right)\bar{g} = 0. \tag{S6}$$

Eq. (S6) is simply the Bessel differential equation, whose solution for ${\omega_n}^2 < M^2$ are Bessel functions $\bar{g} = CJ_L(\omega_n\rho)$ for the region containing $\rho = 0$ and modified Bessel functions (the Bessel functions for complex arguments) $\bar{g} = AK_L\left(\sqrt{M^2 - {\omega_n}^2}\rho\right)$ outside the circle. The ratio of coefficients C/A and frequency ω are found from the continuity boundary condition at $\rho = \mathcal{R}$:

$$C\begin{pmatrix} J_L(\omega_n\mathcal{R}) \\ \frac{1}{\omega_n}\left(\frac{d}{d\rho} + \frac{L}{\rho}\right)J_L(\omega_n\rho)\Big|_{\rho=\mathcal{R}} \end{pmatrix} = A\begin{pmatrix} K_L\left(\sqrt{M^2 - {\omega_n}^2}\mathcal{R}\right) \\ \frac{1}{\omega_n - M}\left(\frac{d}{d\rho} + \frac{L}{\rho}\right)K_L\left(\sqrt{M^2 - {\omega_n}^2}\rho\right)\Big|_{\rho=\mathcal{R}} \end{pmatrix}. \tag{S7}$$

Thus, the dispersion relation is given by the implicit relation

$$\omega_n J_L(\omega_n\mathcal{R})\left(\frac{d}{d\rho} + \frac{L}{\rho}\right)K_L\left(\sqrt{M^2 - {\omega_n}^2}\rho\right)\Big|_{\rho=\mathcal{R}} = (\omega_n - M)K_L\left(\sqrt{M^2 - {\omega_n}^2}\mathcal{R}\right)\left(\frac{d}{d\rho} + \frac{L}{\rho}\right)J_L(\omega_n\rho)\Big|_{\rho=\mathcal{R}}, \tag{S8}$$

$$\sqrt{\frac{M + \omega_n}{M - \omega_n}}J_L(\omega_n\mathcal{R})K_{L-1}\left(\sqrt{M^2 - {\omega_n}^2}\mathcal{R}\right) = K_L\left(\sqrt{M^2 - {\omega_n}^2}\mathcal{R}\right)J_{L-1}(\omega_n\mathcal{R})\,. \tag{S9}$$

The typical solutions of Eq. (S9) are shown in Fig. S1 for different values of $\mathcal{R}$, which is consistent with the numerical solutions of the more advanced continuum model in Fig. S2.

The approximate continuum Dirac model yields a quantized spectrum of doubly degenerate bound modes with $L_\downarrow = -L_\uparrow$. Small degeneracy lifting that is observed in both full-wave eigenmode simulations for specific levels and tight-binding model [see Fig. S3(B)] can be correctly reproduced in the continuum approximation by introducing coupling of the two 2×2 pseudospin sectors $\hat{H}_{\uparrow\downarrow}$ via off-diagonal 2×2 matrices diag$\{Ak_+^2, -Ak_-^2\}$, which was originally neglected in model in Eq.(S1), but can be treated as a perturbation.

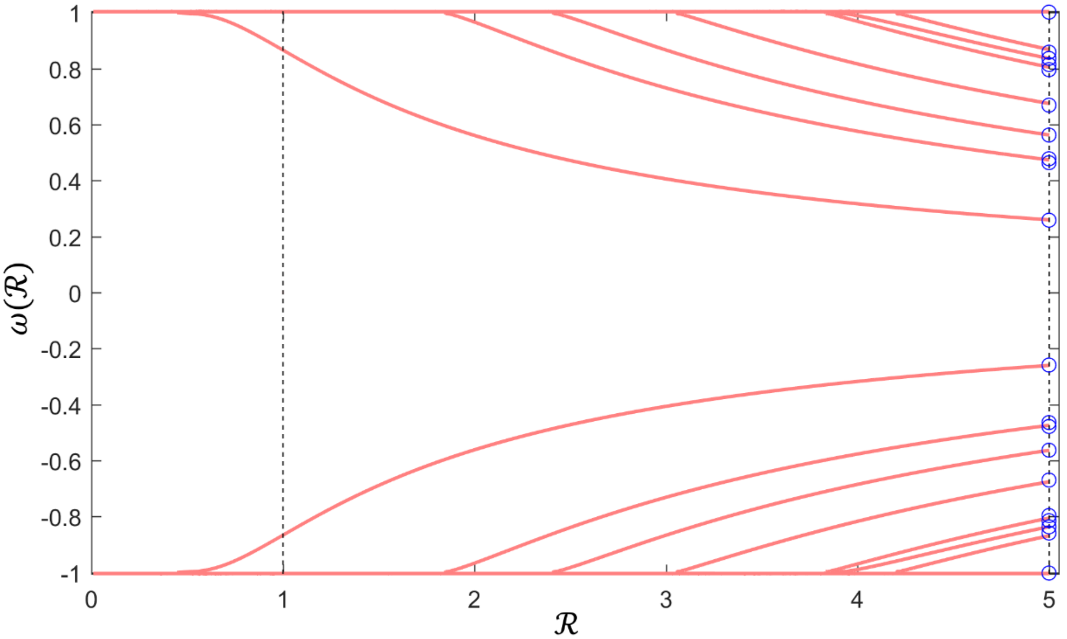

Fig. S1. Spectrum of the Dirac atom. The spectrum $\omega(\mathcal{R})$ for $L = -6:1:7$; $M = 1$: analytical (lines) vs numerical (dots).

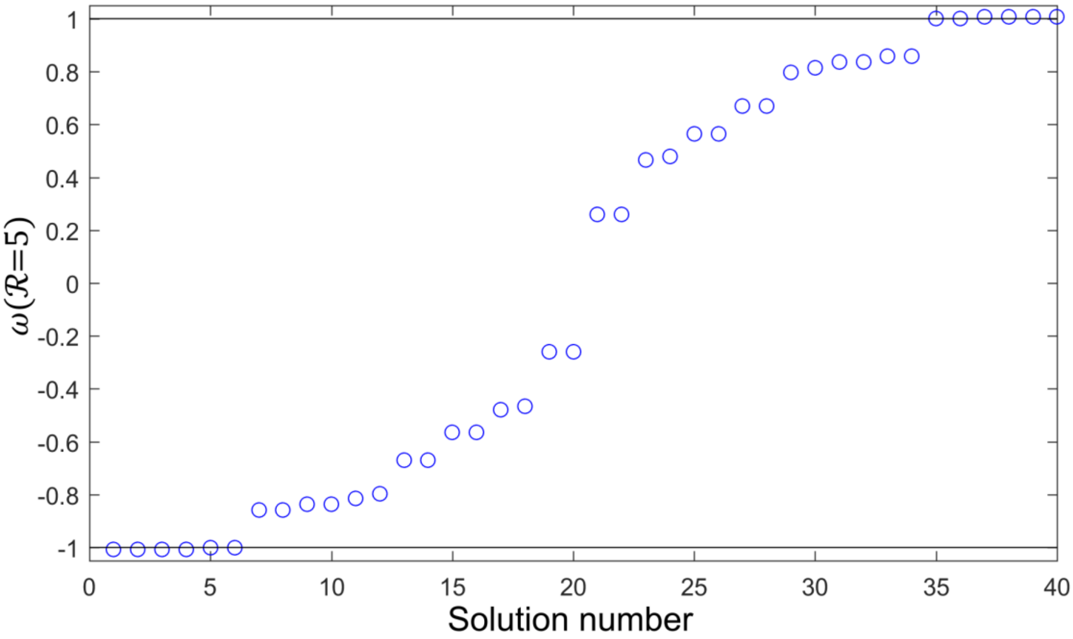

Fig. S2. Spectrum of the Dirac atom with fixed $\mathcal{R} = 5$. The spectrum showing slight degeneracy lifting of some of the levels when spins are weakly coupled due to off-diagonal quadratic term $diag\{Ak_+^2, -Ak_-^2\}$.

Section 2: Supersymmetry in the continuum model

Certain states of the continuum Dirac equation can be found by using the supersymmetric technique [52]. We assume the wavefunction for the two-dimensional Dirac equations has the form

$$\boldsymbol{\psi}_{\uparrow/\downarrow} = \frac{1}{\sqrt{2\pi\rho}}\begin{pmatrix} f_L(\rho)e^{\pm i\varphi} \\ \mp i g_L(\rho) \end{pmatrix} e^{iL\varphi} \equiv \frac{1}{\sqrt{2\pi\rho}}\begin{pmatrix} \phi_1(\rho)e^{\pm i\varphi} \\ \phi_2(\rho) \end{pmatrix} e^{iL\varphi} \tag{S10}$$

We solve the eigenvalue problem for the spin-down block, $\widehat{H}_{\downarrow}\boldsymbol{\psi} = \omega_n\boldsymbol{\psi}$. Substituting Eq. (S10) into the time evolution equation with Hamiltonian Eq. (S1), we obtain

$$\begin{pmatrix} m(\rho) - \omega_n & -i\left(\partial_\rho + \frac{J}{\rho}\right) \\ -i\left(\partial_\rho - \frac{J}{\rho}\right) & -m(\rho) - \omega_n \end{pmatrix}\begin{pmatrix} \phi_1(\rho) \\ \phi_2(\rho) \end{pmatrix} = 0\,. \tag{S11}$$

where $J = L - \frac{1}{2}$. Taking the derivative in the Eq. (S11) leads to

$$\partial_\rho^2\phi_1 = \left(\frac{J(J-1)}{\rho^2} + m^2 - {\omega_n}^2\right)\phi_1 + i\partial_\rho m\,\phi_2, \tag{S12}$$

$$\partial_\rho^2\phi_2 = \left(\frac{J(J+1)}{\rho^2} + m^2 - {\omega_n}^2\right)\phi_2 - i\partial_\rho m\,\phi_1. \tag{S13}$$

This is Klein-Gordon (KG) equation for spin-up components. Let $w_{\mp} = \phi_1 \mp i\,\phi_2$, in terms of which the KG equation becomes

$$\partial_\rho^2 w_+ = \left(\frac{J^2}{\rho^2} + m^2 + \partial_\rho m - {\omega_n}^2\right) w_+ - \frac{J}{\rho^2} w_-\,, \tag{S14}$$

$$\partial_\rho^2 w_- = \left(\frac{J^2}{\rho^2} + m^2 - \partial_\rho m - {\omega_n}^2\right) w_- - \frac{J}{\rho^2} w_+\,. \tag{S15}$$

Let $(\overline{w}_+, \overline{w}_-)^T = \widehat{U}(w_+, w_-)^T$, where the matrix $\widehat{U}$ diagonalizes the matrix $\partial_\rho m\,\widehat{\sigma}_z - \frac{J}{\rho^2}\widehat{\sigma}_x$, we have

$$\partial_\rho^2\overline{w}_+ = \left(\frac{J^2}{\rho^2} + m^2 + \sqrt{\frac{J^2}{\rho^4} + \left(\partial_\rho m\right)^2} - {\omega_n}^2\right)\overline{w}_+\,, \tag{S16}$$

$$\partial_\rho^2\overline{w}_- = \left(\frac{J^2}{\rho^2} + m^2 - \sqrt{\frac{J^2}{\rho^4} + \left(\partial_\rho m\right)^2} - {\omega_n}^2\right)\overline{w}_-\,. \tag{S17}$$

For the $J = 0$, we have

$$\partial_\rho^2 \bar{w}_- = \left(m^2 - \partial_\rho m - \omega_n{}^2\right)\bar{w}_- \,, \tag{S18}$$

$$\partial_\rho^2 \bar{w}_+ = \left(m^2 + \partial_\rho m - \omega_n{}^2\right)\bar{w}_+ \,. \tag{S19}$$

Defining the operators

$$\hat{a} = \partial_\rho + m \Rightarrow \ \hat{a}^+ = -\partial_\rho + m \,, \tag{S20}$$

KG equation (S18), (S19) is rewritten as

$$(\hat{a}^+\hat{a} - \omega_n{}^2)\bar{w}_- = 0 \,, \tag{S21}$$

$$(\hat{a}\hat{a}^+ - \omega_n{}^2)\bar{w}_+ = 0 \,. \tag{S22}$$

For $\omega_n = 0$, we have $\hat{a}^+\hat{a}\,\bar{w}_- = 0 \Rightarrow \ \hat{a}\bar{w}_- = 0$. Therefore, we have the zero-energy solution for $J = 0$, i.e.,

$$\bar{w}_-(\rho) = ce^{-\int_0^\rho m(r)dr}. \tag{S23}$$

For the case of Gaussian mass term, $m(\rho) = \alpha\left(1 - \ e^{-(\rho/\sigma)^2}\right)$, we obtain

$$\bar{w}_-(\rho) = ce^{-\alpha\left(\rho - \sigma\frac{\sqrt{\pi}}{2}\mathrm{erf}(\rho/\sigma)\right)}. \tag{S24}$$

By using the creation and annihilation operator, we could rewrite the effective Hamiltonian with pseudospin down in the form of

$$\hat{H}_\downarrow = \frac{1}{2}\begin{bmatrix} \hat{a}^+\hat{a} & 0 \\ 0 & \hat{a}\hat{a}^+ \end{bmatrix} = \frac{1}{2}\{\hat{Q}^+, \hat{Q}\}, \tag{S25}$$

where the supercharge operator $\hat{Q} \equiv \hat{a} \otimes \begin{bmatrix} 0 & 0 \\ 1 & 0 \end{bmatrix}$, and satisfy the restriction $\hat{Q}^2 = \hat{Q}^{+^2} = 0$.

We could express the KG equation as

$$\hat{H}_\downarrow \begin{pmatrix} \bar{w}_- \\ \bar{w}_+ \end{pmatrix} = \omega_n{}^2 \begin{pmatrix} \bar{w}_- \\ \bar{w}_+ \end{pmatrix}. \tag{S26}$$

With supersymmetric Hamiltonian

$$\hat{H}_\downarrow = \frac{1}{2}\left(\partial_\rho^2 + m^2(\rho)\right)\hat{\sigma}_0 - \frac{1}{2}m'(\rho)\hat{\sigma}_z \,. \tag{S27}$$

The first factor is a Hamiltonian for a spin polarized particle or hole with unit mass moving on a potential $V(\rho) = m^2(\rho)$. The term comes with 2×2 identity matrix $\hat{\sigma}_0$ and so doesn't care about the particle or hole. In contrast, the second term comes with the Pauli-Z matrix which acts like electric field, distinguishing particle and hole by a minus sign. Based on the supersymmetric quantum mechanics, at each energy level, the number of states must be even. But in our model, that means that there are even numbers of states for each ω^2, half number of these states are particle with ω, other states are hole with $-\omega_n$.

Except the Gaussian mass term, we can choose other type of the mass term, for example $m(\rho) = \tanh(\rho/\delta)$, $\delta > 0$. The KG equation for $J = 0$ becomes a Schrodinger equation with the Pöschl-Teller potential:

$$\left(-\partial_\rho^2 + \tanh^2\left(\frac{\rho}{\delta}\right) - \frac{1}{\delta}\operatorname{sech}^2\left(\frac{\rho}{\delta}\right) - {\omega_n}^2\right)\overline{w}_- = 0\,. \tag{S28}$$

By substituting $z = \tanh(\rho/\delta)$,

$$\left[\partial_z\left((1-z^2)\partial_z\right) + \delta(1+\delta) - \frac{\mu^2}{1-z^2}\right]\overline{w}_- = 0, \tag{S29}$$

where $\mu \equiv \delta\sqrt{1-{\omega_n}^2}$.

The general solution of Eq. (S30) is given by

$$\overline{w}_- = c_1 P_\delta^\mu(z) + c_2 Q_\delta^\mu(z), \tag{S30}$$

where P_δ^μ and Q_δ^μ denote the associated Legendre functions of the first and second kind, respectively.

$${\omega_n}^2 = 1 - \frac{\mu^2}{\delta^2}, \qquad 0 \leq \mu \leq \delta, \tag{S31}$$

where δ and μ are non-negative integers.

Similarly, for the spin-up component $\widehat{H}_\uparrow \boldsymbol{\psi} = \omega_n \boldsymbol{\psi}$, we get

$$\begin{pmatrix} m(\rho) - \omega_n & i\left(\partial_\rho - \frac{J}{\rho}\right) \\ i\left(\partial_\rho + \frac{J}{\rho}\right) & -m(\rho) - \omega_n \end{pmatrix}\begin{pmatrix}\phi_1(\rho) \\ \phi_2(\rho)\end{pmatrix} = 0, \tag{S32}$$

with $J = L + \frac{1}{2}$ and

$$\partial_\rho^2\phi_2 = \left(\frac{J^2 - J}{\rho^2} + m^2 - {\omega_n}^2\right)\phi_2 + i\partial_\rho m\,\phi_1, \tag{S33}$$

$$\partial_\rho^2\phi_1 = \left(\frac{J^2 + J}{\rho^2} + m^2 - {\omega_n}^2\right)\phi_1 - i\partial_\rho m\,\phi_2. \tag{S34}$$

Let $v_\mp = \phi_2 \mp i\phi_1$, in term which the KG equation becomes

$$\partial_\rho^2 v_+ = \left(\frac{J^2}{\rho^2} + m^2 + \partial_\rho m - {\omega_n}^2\right)v_+ - \frac{J}{\rho^2}v_-\,, \tag{S35}$$

$$\partial_\rho^2 v_- = \left(\frac{J^2}{\rho^2} + m^2 - \partial_\rho m - {\omega_n}^2\right)v_- - \frac{J}{\rho^2}v_+\,. \tag{S36}$$

Let $(\bar{v}_+, \bar{v}_-)^T = \widehat{U}(v_+, v_-)^T$, where $\widehat{U}$ diagonalize the matrix $\partial_\rho m\widehat{\sigma}_z - \frac{J}{\rho^2}\widehat{\sigma}_x$, we have

$$\partial_\rho^2 \bar{v}_+ = \left(\frac{J^2}{\rho^2} + m^2 + \sqrt{\frac{J^2}{\rho^4} + \left(\partial_\rho m\right)^2} - {\omega_n}^2\right)\bar{v}_+ \, , \tag{S37}$$

$$\partial_\rho^2 \bar{v}_- = \left(\frac{J^2}{\rho^2} + m^2 - \sqrt{\frac{J^2}{\rho^4} + \left(\partial_\rho m\right)^2} - {\omega_n}^2\right)\bar{v}_- \, . \tag{S38}$$

Thus, we get the similar KG equation for both the spin-up and spin-down components. Therefore, each energy level of the effective Hamiltonian $\widehat{H}_{\text{eff}}$ is two-fold degenerate since the spin-up and spin-down subspaces are decoupled in the lower energy subspace of Hilbert space.

Section 3: Solutions of the tight binding model

In this section, we discuss calculation of eigenstates and eigenvalues in the tight binding model for the Dirac atom with C_6 rotational symmetry. First, we define the ratio of the intercell and intracell hopping amplitudes for each layer of the atom, so that we can recover the effective mass term as a function of layer R_L, which takes the form $m(R_L) = \alpha\left(1 - e^{-(R_L/\beta)^2}\right)$, as shown in Fig. S3(A). Second, we diagonalize the tight binding Hamiltonian to solve the eigenproblem. In Fig. S3(B), we show typical eigenvalues nearby the zero-energy level. We observe several two-fold degenerate eigenstates, e.g., $\mathcal{E}_1$ and $\mathcal{E}_2$, $\mathcal{E}_5$ and $\mathcal{E}_6$, while for some of the pairs the degeneracy is lifted, e.g., $\mathcal{E}_3$ and $\mathcal{E}_4$. This degeneracy breaking is because pseudospin up and pseudospin down states are weakly coupled in the tight binding model even for the lower energy subspace, cf. Fig. S2. The amplitude distributions of typical eigenstates are shown in Fig. S4.

The energy levels can be assigned orbital quantum numbers, in qualitative agreement with the spin-degenerate solutions of the continuum Dirac model. As described above, the amplitude distributions of eigenstates with negative energies are readily obtained by applying the substitution $\mathcal{E}_n \to (-\mathcal{E}_n)$ and $L_\downarrow \to (1 - L_\downarrow)$. There may be several modes with the same $L_{\uparrow/\downarrow}$ but different radial distribution which depending on the trapping cavity size. Like a hydrogen atom problem, these distinct radial solutions can be labeled by an additional quantum number n, which is simply the number of nodes in the orbital in the radial direction.

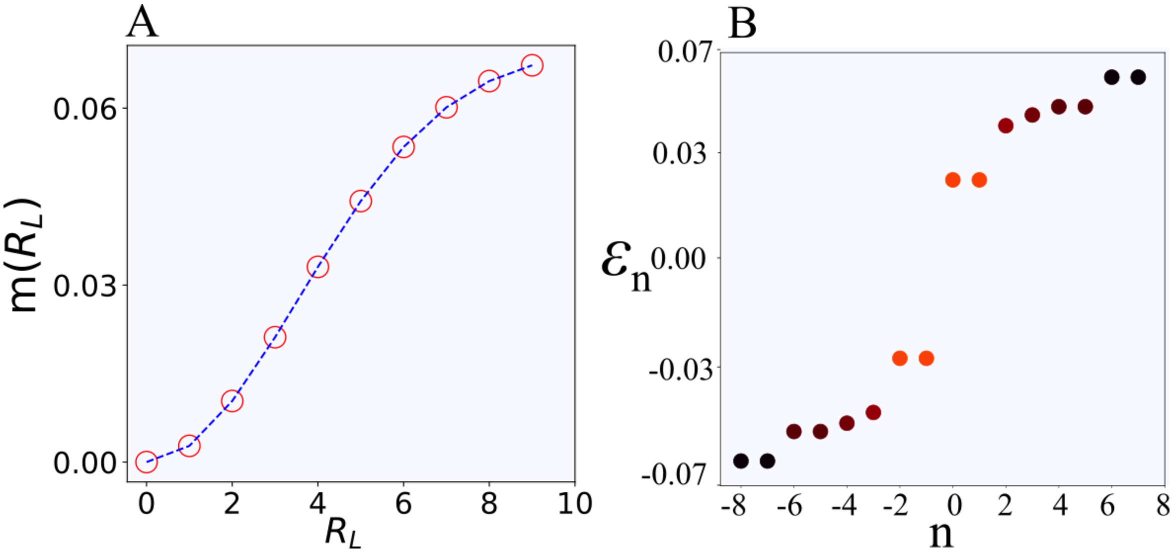

Fig. S3. Mass term profile and spectrum in the tight-binding model. (A) Effective mass term as a function of layer R_L, $m(R_L) = \alpha\left(1 - e^{-(R_L/\beta)^2}\right)$, where $\alpha = 0.07$ and $\beta = 5$. (B) Typical eigenvalues.

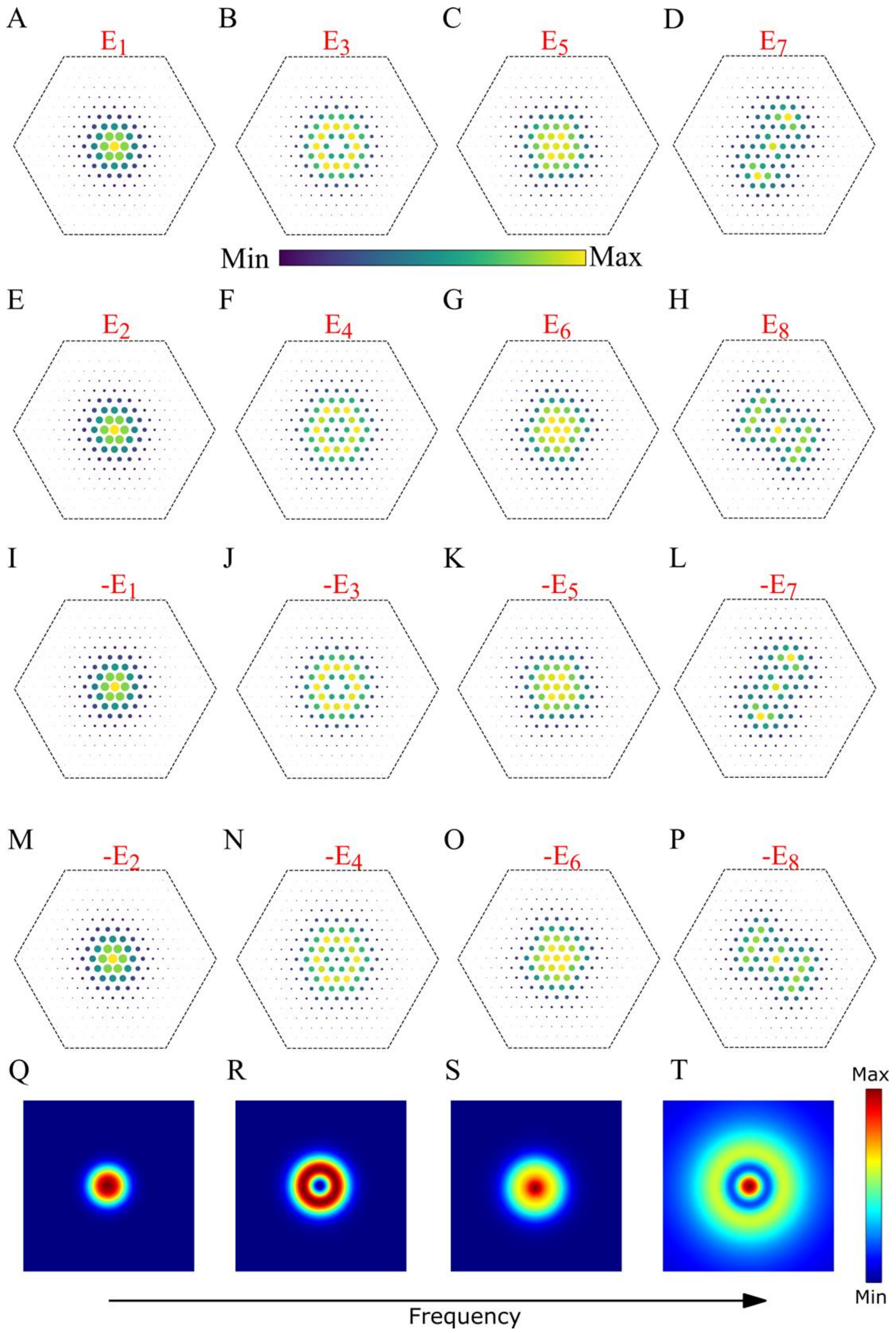

Fig. S4. Orbitals of the Dirac cavity. Typical intensity distributions of trapped eigenstates calculated by using the tight binding model (with eigenenergy, i.e. eigenfrequency, shown in Fig. S3(B)) and continuum Dirac equation (bottom row) with the Gaussian-shape mass term: (Q) first order ($n = 1$), $L_{\downarrow} = 1$, (R) first order ($n = 1$), $L_{\downarrow} = 2$, (S) first order ($n = 1$), $L_{\downarrow} = 0$, (T) second order ($n = 2$), $L_{\downarrow} = 1$. Panels (Q-T) correspond to the pairwise eigenvalues in spectrum Fig. S3(B) above zero. The frequency increases from left to right.

Section 4: Mode profiles of the experimental structure.

In this section, we present a full table of the experimentally observed modes in Fig. S5.

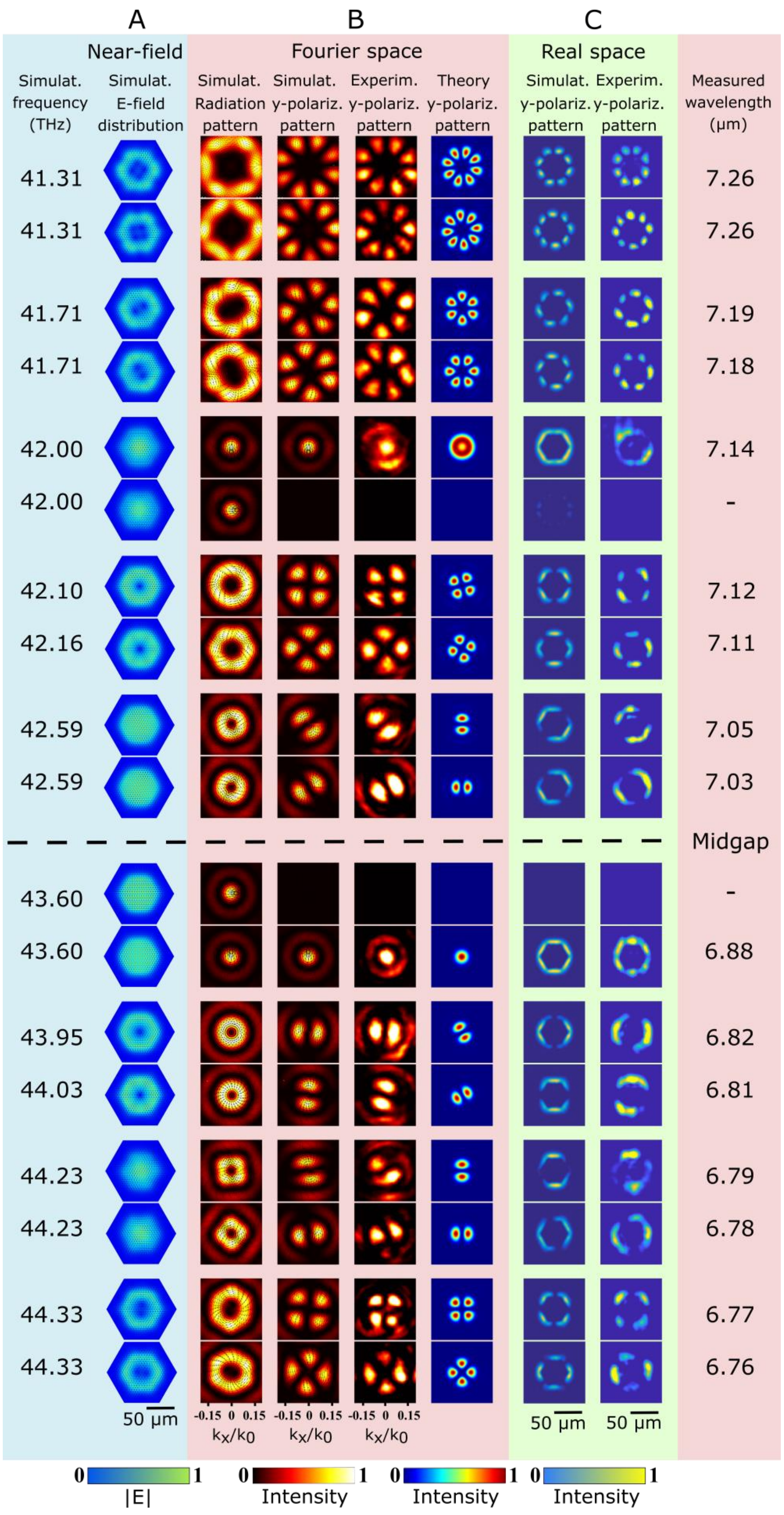

Fig. S5. Modes profiles of the experimental structure. (A) Electric field distribution in eigenmodes of the photonic Dirac atom calculated by first principle simulations with the corresponding frequencies. Mode profiles with negative detuning from the midgap frequencies are similar to the mode profiles with the positive detuning due to the particle-hole symmetry of the designed photonic Dirac atom. (B, C) Mode profiles of the fabricated structure in Fourier and real space.

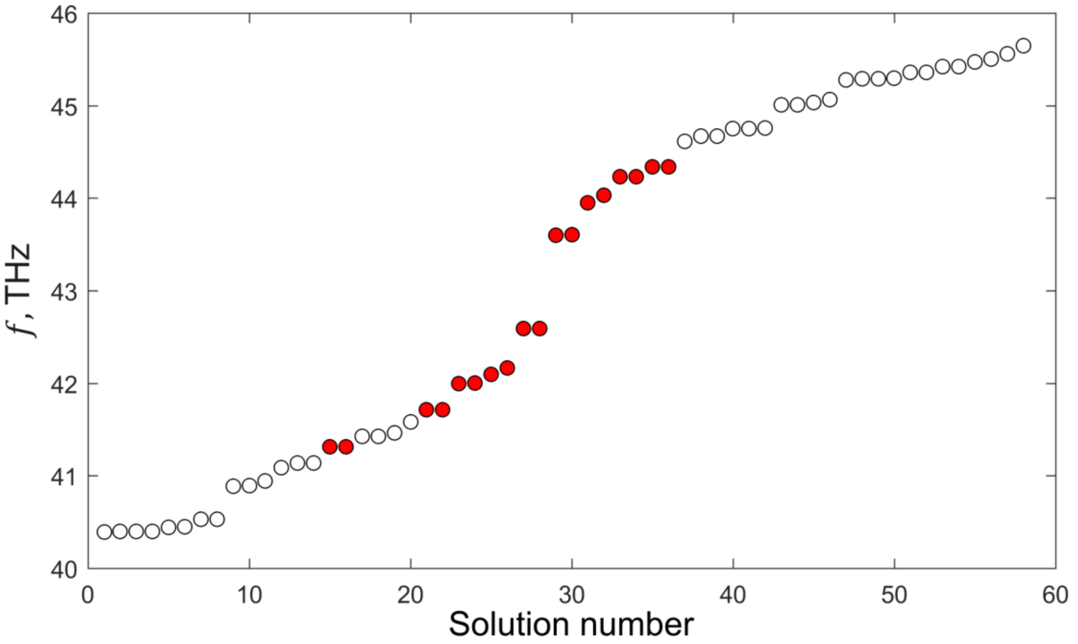

Fig. S6. Spectrum of eigenmodes obtained using full-wave numerical simulations. Red dots correspond to the modes shown in Fig.S5.

Section 5: Comparison of different mass term profiles – smooth vs sharp.

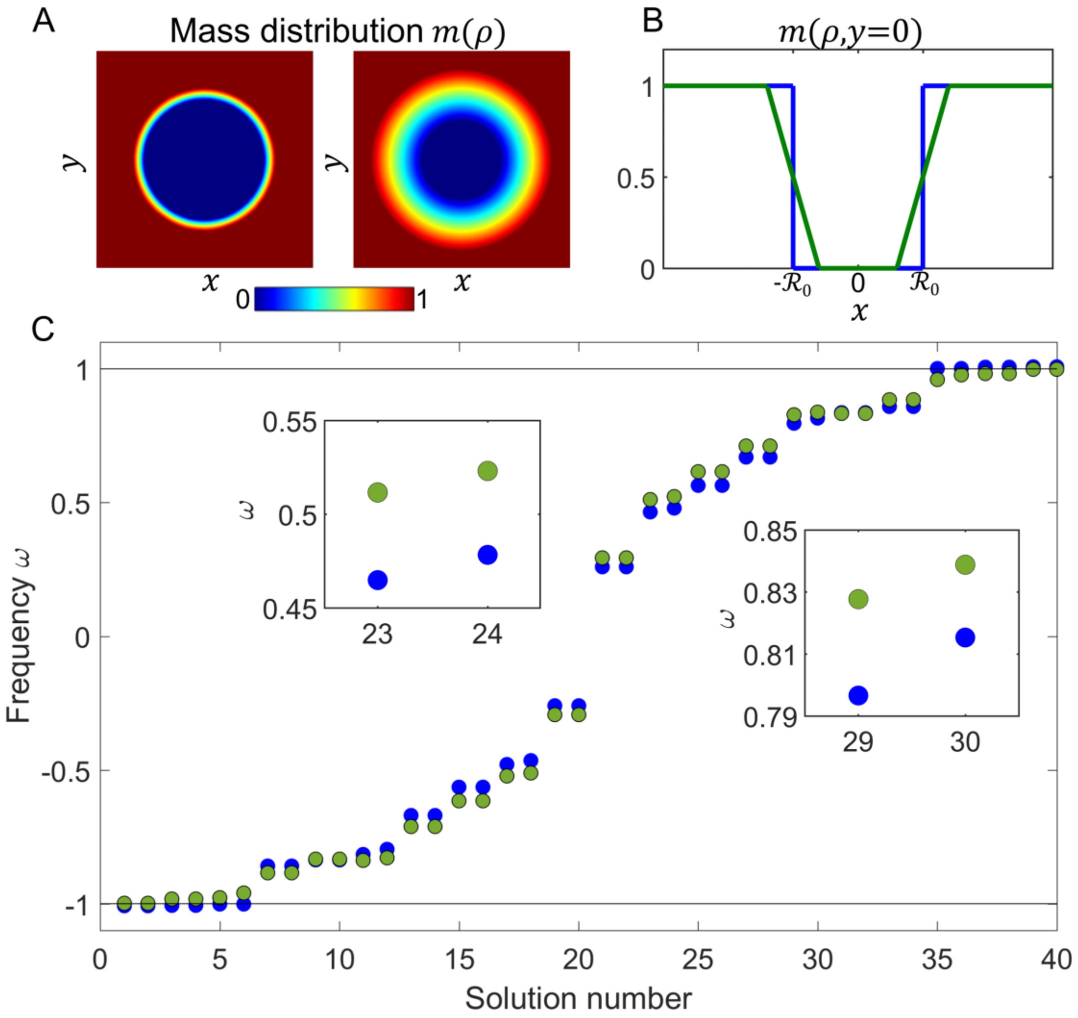

Fig. S7. **Comparison of the solutions of the continuous model with different mass term profiles: step mass function and linear function of the transition layer.** (A, B) Mass term profiles. (C) Spectra of two models with different mass terms: blue dots correspond to the mass function, green dots correspond to the linear mass function. Inserts show decrease of eigenvalues splitting in the model with a smooth transition: the relative frequency shift $\Delta\omega/\omega_m$ decrease from 2.9% to 2.2% for the second pair above zero frequency shown on the left and from 2.3% to 1.3% for the fourth pair above the zero frequency shown on the right.

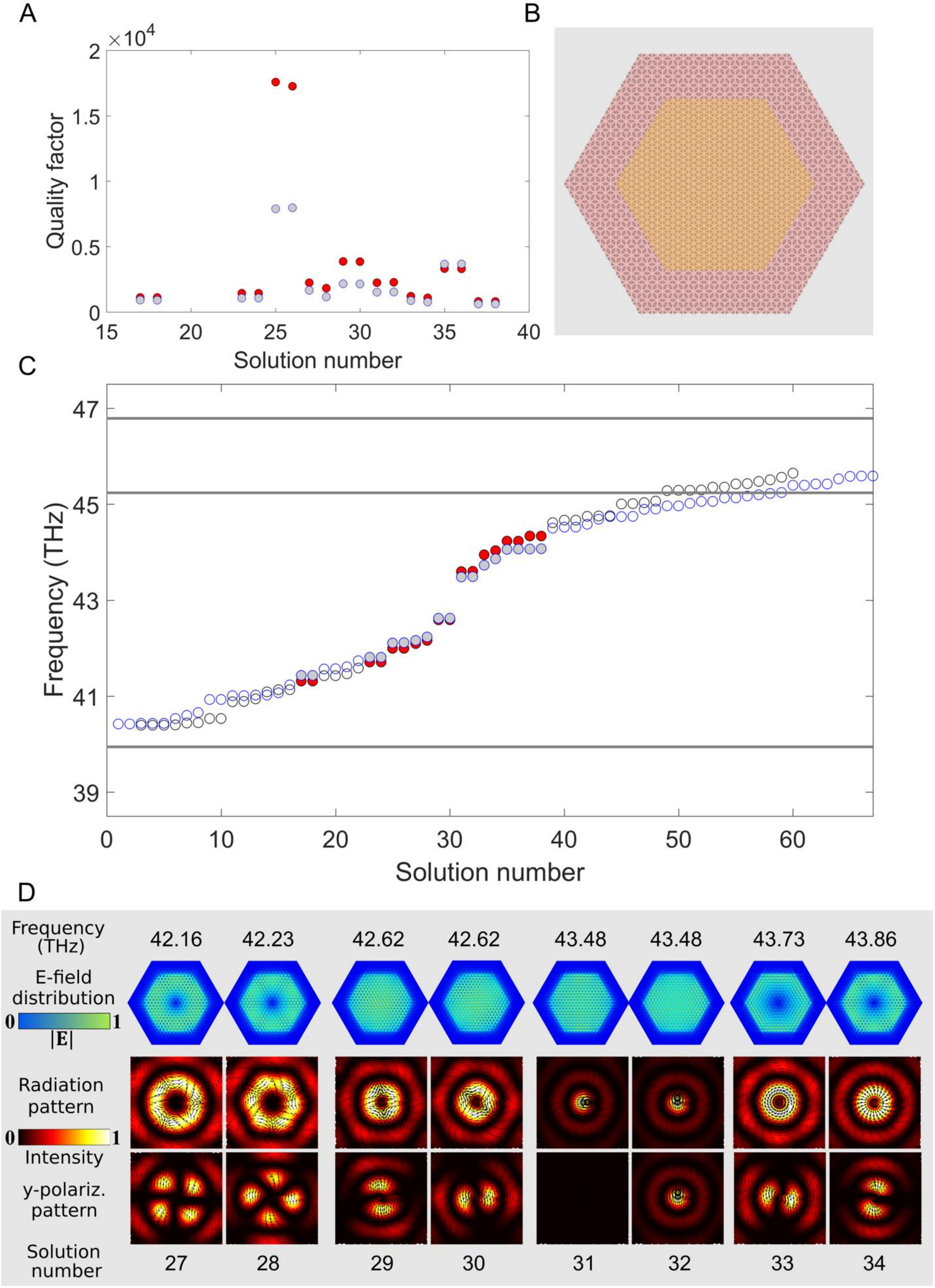

A
×10^4
Quality factor
Solution number
B
C
Frequency (THz)
Solution number
D
Frequency (THz)
42.16
42.23
42.62
42.62
43.48
43.48
43.73
43.86
E-field distribution
0
1
|E|
Radiation pattern
0
1
Intensity
y-polariz. pattern
Solution number
27
28
29
30
31
32
33
34

Fig. S8. **Quality factors, spectrum, and electric field distributions of eigenmodes.** (A) Comparison of the quality factors of the eigenmodes of the experimental structure and structure with a step-wise variation of unit cells. Red dots correspond to eigenmodes shown in Fig. S5, lilac dots correspond to the same eigenmodes of the step-wise structure. (B) Sketch of the photonic Dirac atom with a step-wise variation of unit cells: the unperturbed unit cells inside the cavity and the expanded unit cells outside. (C) Spectrum of eigenmodes obtained using full-wave numerical simulations. Black circles correspond to the modes of the experimental structure; lilac circles correspond to the modes of step-wise structure. Black solid lines correspond to the band gap of the periodic structure with the maximally expanded unit cell shown in Fig. S9A. (D) Electric field distributions and profiles in the Fourier space of the eigenmodes with solution numbers 27-34 of the step-wise Dirac cavity shown in (B).

Section 6: Simulations of the effect of sharp corners on the band structure.

We have performed simulations of the effect of sharp corners on the band structure. We have found that rounding of less than 20% (radius of curvature at corners/triangle side length<0.2), there is little to no effect on the band structure. Larger values of rounding, however, give rise to penetration of the undesirable band into the region of band gap open by the lattice perturbation. These conclusions agree with the results of the paper by Barik et al.[18], which was the first to propose triangular shapes for complete topological bandgap. Figures S9 A and B show two cases corresponding to the perfectly triangular shapes (as in Fig. S9 C) and rounded triangles with degree of rounding found in experimental samples (as in Fig. S9 D), respectively. It is clear from the band structure comparison that the effect of rounding on the photonic band structure for this case is negligible.

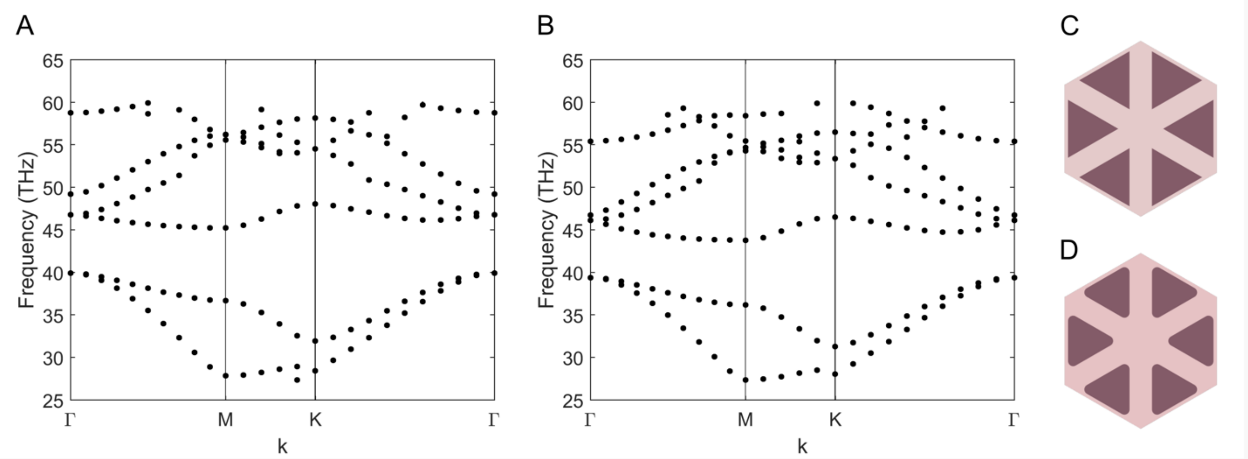

Fig. S9. **Simulated photonic band structure of uniform metasurfaces.** (A) Band structure of metasurface with unit cells which have holes with perfect sharp corners. (B) Band structure for unit cells with holes with the round corners. (C, D) Sketches of expanded unit cells with different corners of triangle holes.
